# Serological evidence for transmission of multiple dengue virus serotypes in Papua New Guinea and West Papua prior to 1963

**DOI:** 10.1371/journal.pntd.0005488

**Published:** 2017-04-24

**Authors:** Dagwin Luang-Suarkia, Timo Ernst, Michael P. Alpers, Ralph Garruto, David Smith, Allison Imrie

**Affiliations:** 1 School of Biomedical Sciences, University of Western Australia, Nedlands, Western Australia, Australia; 2 Virology Laboratory, Papua New Guinea Institute of Medical Research, Goroka, Papua New Guinea; 3 Curtin University, Shenton Park, Western Australia, Australia; 4 Binghamton University, Binghamton, New York, United States of America; 5 Pathwest Laboratory Medicine WA, Nedlands, Western Australia, Australia; Oregon Health and Science University, UNITED STATES

## Abstract

Little is known about the natural history of dengue in Papua New Guinea (PNG). We assessed dengue virus (DENV)-specific neutralizing antibody profiles in serum samples collected from northern and southern coastal areas and the highland region of New Guinea between 1959 and 1963. Neutralizing antibodies were demonstrated in sera from the northern coast of New Guinea: from Sabron in Dutch New Guinea (now known as West Papua) and from four villages in East Sepik in what is now PNG. Previous monotypic infection with DENV-1, DENV-2, and DENV-4 was identified, with a predominance of anti-DENV-2 neutralizing antibody. The majority of positive sera demonstrated evidence of multiple previous DENV infections and neutralizing activity against all four serotypes was detected, with anti-DENV-2 responses being most frequent and of greatest magnitude. No evidence of previous DENV infection was identified in the Asmat villages of the southern coast and a single anti-DENV-positive sample was identified in the Eastern Highlands of PNG. These findings indicate that multiple DENV serotypes circulated along the northern coast of New Guinea at different times in the decades prior to 1963 and support the notion that dengue has been a significant yet neglected tropical infection in PNG for many decades.

## Author summary

Dengue is a mosquito-borne disease caused by infection with any of the four dengue virus serotypes (DENV-1 –DENV-4), which are transmitted in more than 100 tropical and subtropical countries. The current global dengue burden, and dengue mortality, is greatest in the southeast Asian and western Pacific region where more than 70% of people at risk of infection reside. All four DENV serotypes have been reported to circulate in this region and each DENV serotype has been associated with high rates of morbidity and mortality. Sequential infection with heterologous DENV serotypes is associated with more severe dengue disease (previously known as dengue hemorrhagic fever and dengue shock syndrome) and co-circulation of multiple DENV serotypes is frequently observed in endemic countries. Substantial variation in local capacity for systematic surveillance and reporting among countries in the region means dengue burden is likely underestimated. We tested archival serum samples collected more than 50 years ago in Papua New Guinea in order to begin to assess the true burden of dengue, in a country where severe dengue has not been reported and DF is rare. Serological evidence for previous monotypic and multitypic DENV infection in adults living along the northeastern coast of PNG between 1959–1963 indicates dengue was transmitted prior to this period. The contribution of dengue to acute febrile illness in PNG, and the reasons for the apparent lack of severe disease, should be investigated.

## Introduction

Dengue is a mosquito-borne disease of humans caused by infection with the dengue viruses (DENV). An estimated 390 million infections occur each year in tropical and subtropical countries of which about 98 million are symptomatic; in endemic countries dengue is associated with significant morbidity and mortality. The frequency, magnitude, and geographic range of dengue epidemics began to increase dramatically after the Second World War when major demographic and ecological changes resulted in increased transmission of the dengue viruses and the disease is now endemic in more than 100 tropical and subtropical countries [1–3]. Incidence has increased consistently in Southeast Asia and the Western Pacific in the last decade, and more than 70% of the global dengue disease burden is currently borne by people who live in this region [4].

Dengue is caused by infection with any one of four dengue viruses (DENV), RNA viruses which form their own antigenic complex within the Flaviviridae family [5]. The four serotypes, DENV-1 –DENV-4, are further classified into genetically distinct groups or genotypes with sequence divergence of up to 6% [6]. Infection is believed to confer lifelong immunity to the homologous DENV serotype and short-lived cross-protective immunity to the other three serotypes [7], and people who live in endemic areas may be infected up to four times in their lifetime. Symptomatic DENV infection typically presents as a non-specific acute febrile illness (dengue fever, DF) which usually develops 3–8 days following a bite from an infected *Aedes* mosquito, most commonly *Aedes aegypti* or *Aedes albopictus*. Severe dengue (previously known as dengue hemorrhagic fever, DHF, and dengue shock syndrome, DSS) is an acute vascular permeability syndrome which occurs in a subset of patients with DF; mortality rates in severe dengue range between 2.5%-20% [8]. The risk of developing severe dengue is greatly increased in a secondary infection with heterologous DENV [9,10].

Dengue transmission has been documented in the Asia-Pacific region since the 1940s when the four prototype DENV were identified in Japan (DENV-1_Mochizuki_,1943), and Hawaii (DENV-1_Hawaii_, 1944), New Guinea (DENV-2_NGC_, 1944), and the Philippines (DENV-3_H-87_ and DENV-4_H-241_, 1956) [11–13]. DENV-2_NGC_ was isolated from US soldiers who fought in New Guinea in 1942–1944 [14]. US Military records indicate there were a large number of suspected infections in New Guinea—approximately 27,000 cases—and several epidemic foci [15]. No severe disease was recorded, however dengue was considered to be a major cause of loss of troop strength. In the years following the Second World War dengue transmission was identified in serosurveys conducted on the island of New Britain in 1971 [16] and in the Bismarck Archipelago in 1975 [17]. More recently an analysis of patients presenting with acute febrile illness in Madang Province on the northern coast of PNG in 2007–2008 identified DENV infection (measured by IgG, IgM, and NS1 antigen) in 8% of cases [18]; no severe dengue was identified in these patients. Although dengue surveillance is not conducted in PNG, genetic analysis of serum samples collected from febrile travelers entering northern Australia from PNG between 1999 and 2010 has identified importation of DENV-1, DENV-2, and DENV-3 [19,20], indicating that multiple serotypes have circulated in recent times.

Dengue epidemics occur frequently in the Asia-Pacific region and DHF/DSS-associated epidemics have been recorded in many countries in this region since the 1950s [4,21,22]. A recent analysis of dengue surveillance data collected between 1968 and 2013 in Indonesia, a country which experiences frequent, large-scale dengue epidemics and shares geographic borders with PNG, found that the incidence of DHF has increased substantially over the past 45 years [23]. Severe dengue has not been reported in PNG despite the likely transmission of multiple DENV serotypes and the potential for introduction of DENV circulating in neighbouring countries such as Indonesia, and the reasons for this are not understood. We undertook a retrospective analysis of DENV seroprevalence in PNG using serum samples collected between 1959 and 1963 in order to identify the DENV serotypes which circulated before that time, and their geographic distribution. These findings will clarify the length of time dengue has been present in PNG, inform our understanding of the natural history of dengue in PNG, and provide a basis for future surveillance and control efforts.

## Materials and methods

### Ethics statement

Ethics approval for this study was granted by the Medical Research Advisory Committee, Ministry of Health, Government of Papua New Guinea; the Institutional Review Board, Binghamton University (54-6-2); and the Human research Ethics Committee, University of Western Australia (RA/4/1/4050).

### Serum samples

Archival human sera were obtained from the National Institutes of Health-National Institute of Neurological Disorders and Stroke (NIH-NINDS) Serum Archive of Binghamton University, New York, USA. Samples were collected in New Guinea (now known as PNG) from East Sepik (1959); Chimbu (1962); and Eastern Highlands (1962) including villages in the hinterlands of present-day Morobe Province (Table 1). Additional samples were collected in Dutch New Guinea (now known as West Papua) in 1962–1963, from the northeast coast Central Highlands and southern coast (Table 2). The samples were stored at –20°C or -80°C at the NIH and Binghamton University prior to analysis.

**Table 1:** Study population, Papua New Guinea.

Origin and year of sampling			Number of samples/gender			Age (years) median (range)	
Year	Region	Village	Male	Female	Total	Male	Female
1959	East Sepik	Suonambo	10	11	21	46(27–72)	41(28–65)
		Arisili	5	6	11	39(25–52)	45(25–58)
		Luwaite	1	1	2	47	37
		Wahlen	7	6	13	37(24–52)	38(24–54)
		Not specified*	4	1	5	NA	65
	Subtotal		27	25	52		
1962	Chimbu	Mul	0	1	1		45
		Moi’o	3	1	4	41.6 (35–50)	25
		Naiyo	1	0	1	35	
		Sogo	2	2	4	25 (25,25)	38(35,40)
		Yawio	0	1	1		23
		Mogiagi	2	0	2	55(50,60)	
	Subtotal		8	5	13		
1962	Eastern Highlands	Wenaio	1	0	1	25	
		Yabibo	4	4	8	40(35–50)	31(25–40)
		Bogiaio	1	5	6	50	40(30–50)
		Huyaio	0	2	2		33(30–35)
		Masi	1	0	1	35	
		Agamusei	2	0	2	31(22,40)	
		Kamaiyana	1	0	1	30	
		So’o	1	1	2	35	35
		U’i	1	0	1	25	
		Wario	1	0	1	40	
		Awarosa	0	1	1		35
		Mugaiamuti	3	0	3	45(40–50)	
		Mobutasa	1	0	1	30	
		Hero	1	0	1	60	
		Sikong	1	0	1	22	
		Po	1	0	1	22	
	Subtotal		20	13	33		
	Total		55	43	98		

*Unnamed village was coded in field notes as originating in East Sepik in 1959. NA: data not available.

**Table 2:** Study population, West Papua.

Origin and year of sampling			Number of samples/gender			Age (years) median (range)	
Year	Region	Village	Male	Female	Total	Male	Female
1962–63	Northeast Coast	Sabron	16	9	25	39(25–60)	34(35–42)
	Subtotal		16	9	25		
1962	Central Highlands	Omba	1	3	4	50	48(40–55)
	Subtotal		1	3	4		
	Southern Coast	Pirimapoen	5	4	9	39(30–55)	36(30–40)
		Sanam	4	0	4	35(30–40)	
		Emine	2	0	2	43(40,45)	
		Waseki	3	1	4	38(30–40)	30
		Aikut	6	0	6	36(30–45)	
		Sinipit	1	1	2	60	30
		Kaibigir	4	1	5	41(35–45)	30
		Kawem	4	5	9	38(30–45)	37(35–40)
		Khogir	1	1	2	50	30
		Ogor	1	0	1	35	
		Asipim	2	3	5	55(55,55)	35(30–45)
		Gomuru	1	0	1	35	
		Kapara	1	0	1	45	
		Javae Kili Mapoi	1	0	1	35	
	Subtotal		36	16	52		
	Total		53	28	81		

### Microneutralization assay

A serum microneutralization (MN) assay was developed, based on approaches described elsewhere [24–26] and used to measure anti-DENV antibodies in the archival serum samples. This approach was selected to allow simultaneous assessment of antibody to all four reference DENV serotypes (DENV-1_Hawaii-2001_; DENV-2_NGC;_ DENV-3_H-87_ and DENV-4_H-241_) in samples with limited volumes (<0.1 mL). Vero cells at a concentration of 5 x 10^5^/mL in in DMEM medium (DMEM (Invitrogen) supplemented with L-glutamine, penicillin and streptomycin) with 10% heat-inactivated FBS (DMEM-10 growth medium) were prepared and 0.1 mL was added to all wells of 96-well flat-bottomed tissue culture plates (BD Falcon) for a final input of 5 x 10^4^ cells/well, and the plates were incubated in a humidified incubator at 37°C in 5% CO_2_ for 48 hours until wells were confluent. Test sera were diluted 1:10 in DMEM medium and heat-inactivated at 56°C for 30 minutes, then serially diluted two-fold to a dilution of 1:1280 in 96-well round-bottom plates. An equal volume of titrated virus was added to the prepared sera, for a final dose of 50 TCID_50_, and the plates were incubated at 37°C in 5% CO_2_ for 1 hour. Growth medium was removed from the Vero cell monolayer and replaced with the serum-virus mixture. Sera were tested in duplicate against all four DENV serotypes simultaneously. Plates were incubated in a humidified 37°C incubator in 5% CO_2_ for 9–10 days during which time cell monolayers were monitored; appearance of the cells was compared to media-only wells on the same plates. At the end of the incubation culture supernatant was removed, cells were fixed in cold PBS:acetone (1:1), and virus replication was assessed by fixed-cell ELISA. Absence of infectivity, indicated by OD_415/490_ values approaching background (cell-only control well) levels, indicated the presence of DENV-specific antibodies in the serum sample and thus constituted a positive neutralization reaction. The assay cutoff value for neutralization was established as the mean OD_415/490_ of serum only (no virus) control wells plus 2 standard deviations. Wells with higher values were deemed negative for neutralization and the neutralization titer was the reciprocal of the highest serum dilution with a mean OD below the cutoff value. OD_415/490_ for cell-only control wells was <0.3 and for virus control wells was >1.0. Data were presented as Geometric Mean Titers (GMT).

Standard anti-DENV-1-4 sera NIBSC 05/248 (National Institute for Biological Standards and Control [NIBSC], Potter’s Bar, Hertfordshire, United Kingdom) tested against homologous reference DENV consistently produced MN titers of 10–20 (S1 Table) and thus the cutoff value for a positive test result was a titer (reciprocal serum dilution) of 10. Testing of these Standard DENV-1-4 sera in a classical 90% plaque reduction assay [27–28] also produced PRNT-90 values of 10, affirming the sensitivity of the MN assay as utilized in the present study. Cross-neutralization experiments in which Standard DENV-1-4 sera were each tested against heterologous reference DENV consistently produced negative results; each Standard serum was tested against all four DENV reference viruses used in the MN assay (S1 Table).

Serum samples from individuals with well-defined monotypic or multitypic DENV infection [27–29] consistently produced monotypic MN titers greater than 10 to the homologous virus and serum samples from individuals with diagnosed other flavivirus infection (JEV, MVEV, and KUNJV) were tested against DENV-1-4 and were always negative (S1 Table). There was insufficient archival sample volume to perform MN tests against JEV, MVEV and KUNV. Serum samples from 20 individuals with no history of DENV infection, designated as negative controls, who were anti-DENV seronegative by NS1 ELISA (Platelia Dengue NS1 Antigen ELISA kit; Bio-Rad, Australia) and hemagglutination inhibition (HI) assay (PathWest Laboratory Medicine WA) consistently produced MN titers less than 10.

## Results

### Study population

A total of 179 serum samples collected between 1959 and 1963 were analyzed. Sera from 108 males and 71 females were collected from villages and hamlets in PNG (formerly Papua and New Guinea) (98 sera) between 1959 and 1962 and West Papua (formerly Netherlands (Dutch) New Guinea) (81 sera) between 1962 and 1963. Field notes from the time of collection identified three geographic regions in Papua and New Guinea: East Sepik (52 sera), Chimbu (13 sera), and Eastern Highlands (33 sera); and three geographic regions in Dutch New Guinea (West Papua): Northeast coast (25 sera), Central Highlands (4 sera), and Southern Coast (52 sera). These data are summarized in Tables 1 and 2.

### Seroprevalence in Papua and New Guinea (PNG)

Anti-DENV neutralizing antibodies were detected in 39 of 98 (40%) sera from PNG; the remaining 59 were seronegative for all four DENV serotypes. All seropositive sera, with the exception of one sample, were from villages in East Sepik. The additional positive sample was from Po village in the southern border area of Eastern Highlands with Morobe, where villagers were known to hunt and maintain gardens at lower altitudes. Since we do not know the travel history of the 22-year-old man from whom the sample was obtained we cannot be sure that he acquired his DENV infection in the southern lowlands.

These data are summarized in Table 3.

**Table 3:** Distribution of dengue seropositive samples, PNG.

Serotype	Origin						
	East Sepik village					Eastern Highlands	
Monotypic	Suonambo	Arisili	Luwaite	Wahlen	N/A*	Po	Total
DENV-1	2	1	0	0	1	0	4
DENV-2	0	0	0	0	1	0	1
DENV-3	0	0	0	0	0	0	0
DENV-4	0	0	0	1	0	0	1
Subtotal	2	1	0	1	2	0	6
Multitypic							
DENV-1,-3	0	0	0	1	0	0	1
DENV-2,-4	1	0	0	0	1	0	2
DENV-1,-2,-3	1	1	0	0	0	0	2
DENV-1,-2,-4	3	0	2	2	2	2	7
DENV-2,-3,-4	0	0	0	1	0	0	1
DENV-1,-2,-3,-4	9	6	0	2	2	1	20
Subtotal	14	7	2	6	3	1	33
Total	16	8	2	7	5	1	39

*Village name not specified

Of the 39 positive samples, a minority (6/39, 15%) demonstrated a monotypic neutralizing antibody response to DENV-1 (4 sera), DENV-2 (1 sample), or DENV-4 (1 sample). The remaining 33/39 sera (85%) neutralized multiple DENV serotypes. Of these 33 samples, 20 (61%) sera neutralized all four DENV, 10 (30%) neutralized 3 serotypes, and 3 (9%) neutralized 2 serotypes. Anti-DENV-2 was detected in 32/33 samples closely followed by anti-DENV-1 (30 sera) and anti-DENV-4 (30 sera) with anti-DENV-3 positive sera least frequent (24 sera). Among DENV-seropositive sera anti-DENV-2 titers were generally highest with titers up to 160 and DENV-2 GMT of 24.2 was higher than for the other serotypes. In comparison, responses for the other three serotypes demonstrated lower magnitude titers with anti-DENV-1 GMT of 16.3; anti-DENV-3 GMT 18.9; and anti-DENV-4 MN GMT 17.1. (Table 4).

**Table 4:** Dengue neutralizing antibody responses, Papua New Guinea.

Serotype	Number positive	Geometric Mean Titer (range)*			
Monotypic		DENV-1	DENV-2	DENV-3	DENV-4
DENV-1	4	10			
DENV-2	1		10		
DENV-3	0				
DENV-4	1				10
Subtotal	6				
Multitypic					
DENV-1,-3	1	10		20	
DENV-2,-4	2		20		10
DENV-1,-2,-3	2	14.1(10–20)	28.3(20–40)	20(10–40)	
DENV-1,-2,-4	7	10	13.5(10–20)		12.2(10–20)
DENV-2,-3,-4	1		40	10	10
DENV-1,-2,-3,-4	20	22.2(10–80)	30.3(10–160)	19.3(10–80)	21.4(10–80)
Subtotal	33	30	32	24	30
Total	39	34	33	24	31
Overall GMT(range)		16.3(10–80)	24.2(10–160)	18.9(10–80)	17.1(10–80)

* GMT was calculated for sera with MN titers ≥10

Among the East Sepik villages seroprevalence rates were highest for Suonambo (16/21; 76%), Arisili (8/11; 73%) and Wahlen (7/13; 54%), which between them contributed 79% (31/39) of positive sera.

### Dengue seroprevalence in Dutch New Guinea (West Papua)

Sera were available from 2 main areas: the northeastern coast around Hollandia (now known as Jayapura) in West Papua, and the southern coast to the west of the PNG-West Papua border along the Arafura Sea, including Omba village in the hinterlands of the Central Highlands. In total 81 sera were tested including 52 from 14 villages on the southern coast, 25 from the village of Sabron on the northeastern coast, and 4 from Omba. These data are summarized in Table 2.

Anti-DENV antibody was detected exclusively in 20 of 25 sera (80%) collected in Sabron on the northeast coast. These sera accounted for 25% (20/81) of West Papuan samples. Sabron serum samples showed monotypic anti-DENV-2 (9/20; 45%) and anti-DENV-4 (1/20; 5%) responses, with the remaining 10 of 20 (50%) positive samples showing evidence of multiple previous infections: 3 sera neutralized all 4 DENV serotypes, 5 sera neutralized 2 serotypes, and the remaining 2 sera neutralized 3 serotypes. Anti-DENV-2 responses were identified most frequently, with 19 of the 20 positive sera showing some evidence of previous infection with this serotype. Anti-DENV-2 GMT was 16.1, whereas anti-DENV-1 GMT was higher (21.8) despite the lack of monotypic anti-DENV-1 neutralizing capacity. Lower magnitude responses were seen for the 8/20 sera with anti-DENV-4 responses where MN titers were always 10, regardless of the responses detected to other serotypes. Anti-DENV-3 responses were the least frequent and were detected (with MN titers of 10 or 20) in the 3 serum samples which were positive for all 4 serotypes, suggesting that this anti-DENV-3 response was cross-neutralization by antibody to the other 3 prevalent serotypes. The lack of monotypic anti-DENV-3 supports this contention; it does not exclude the possibility that individuals with previous DENV-3 infection were not sampled. These data are summarized in Table 5.

**Table 5:** Dengue neutralizing antibody responses, West Papua.

Serotype	Number Positive	Geometric Mean Titer (range)*			
Monotypic		DENV-1	DENV-2	DENV-3	DENV-4
DENV-1	0				
DENV-2	9		14.6 (10–20)		
DENV-3	0				
DENV-4	1				10
Subtotal	10				
Multitypic					
DENV-1,-2	3	31.7 (20–80)	15.9 (10–40)		
DENV-2,-4	1		10		
DENV-1,-2,-4	2	14.1 (10–20)	20 (10–40)		
DENV-1,-2,-3,-4	3	20 (10–40)	25.2 (20–40)	12.6	10
Subtotal	10	8	10	3	7
Total	20	8	19	3	8
Overall GMT (range)		24.4 (10–80)	16.5 (10–40)	12.6 (10–20)	10

* GMT was calculated for sera with MN titers ≥10

**Fig 1:**
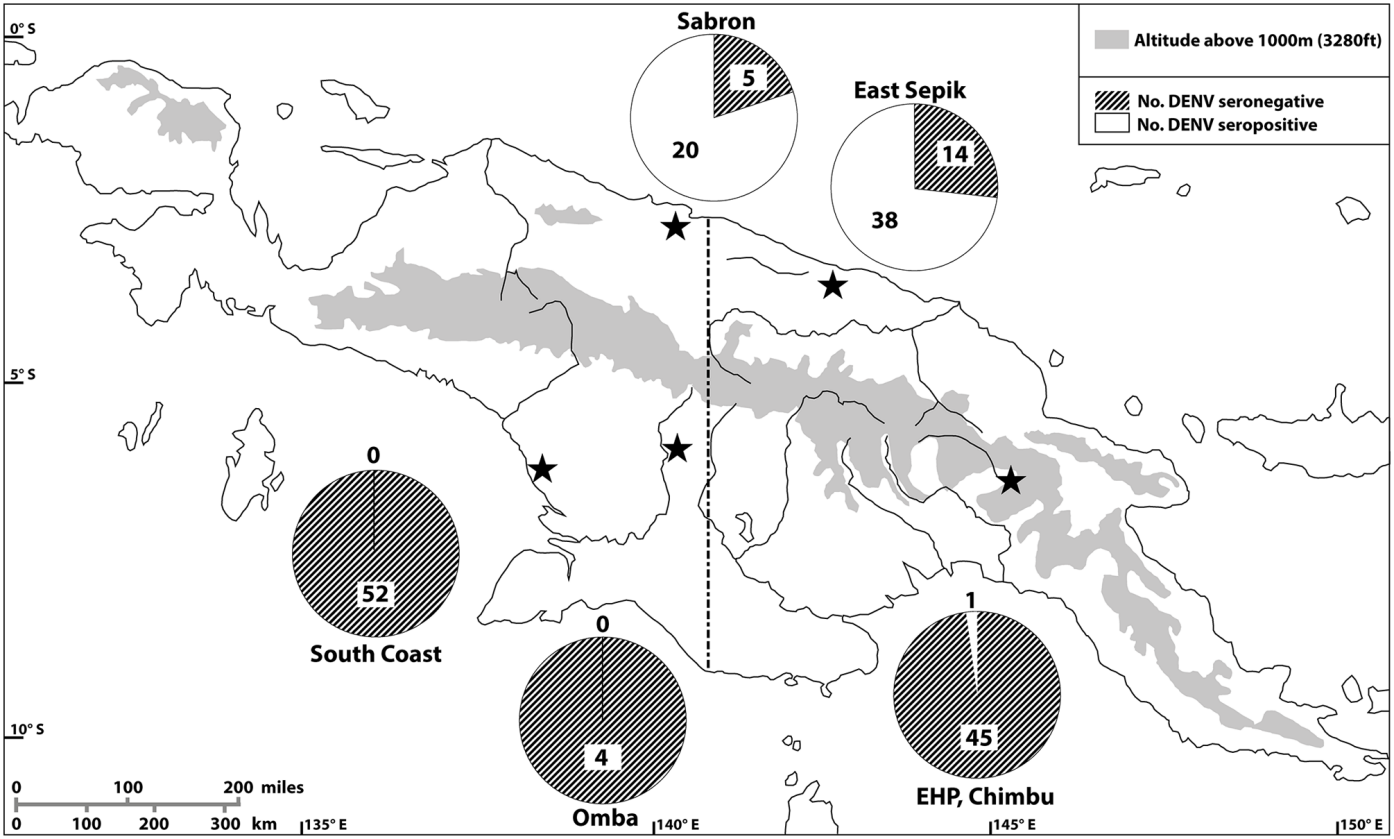
DENV seroprevalence, Papua New Guinea and West Papua 1959–1963. Previous DENV infection was largely identified in Sabron and East Sepik on the northern coast, with a single case in the Eastern Highlands. All sera from the southern coast were seronegative. Stars indicate origin of serum samples collected between 1959–1963. Circles depict number of sera collected at each site, and proportion of sera anti-DENV seropositive and anti-DENV seronegative. Dashed line represents border between New Guinea (now known as Papua New Guinea) to the east, and Dutch New Guinea (now known as West Papua) to the west.

A summary of the geographic distribution of DENV seroprevalence in Papua New Guinea and West Papua is shown in Fig 1.

## Discussion

We undertook a retrospective analysis of DENV seroprevalence in what is now Papua New Guinea and West Papua using serum samples collected between 1959 and 1963, and our findings suggest that multiple DENV serotypes circulated prior to this time period. DENV seroprevalence was largely restricted to the northern Pacific coast of PNG and West Papua and there was no evidence of previous infection in the villages along the southern Casuarine Coast, or in the Highlands.

Monotypic infection with DENV-1, DENV-2, and DENV-4 was identified in 16 of 59 (27.1%) seropositive individuals. Our findings are consistent with the work done by Sabin [12,14] during the Second World War which led to isolation of DENV-2_NGC_ from febrile soldiers deployed in the areas around the northern coastal town of Hollandia (now called Jayapura, in West Papua). Monotypic DENV-4, which was not isolated until 1956, in the Philippines [13], is an interesting finding and implies there was more widespread circulation of the dengue viruses in Oceania in the period prior to the early 1960s than previously understood. The majority of seropositive sera (43/59; 72.9%) neutralized multiple DENV serotypes, suggesting that most exposed individuals had been infected more than once before the time of sampling.

Assessment of prior DENV infection using serum neutralization assays is subject to well-recognized limitations associated with the relatively high degree of homology among the four DENV serotypes. Anti-DENV antibody epitopes may be unique to each serotype, or they may be shared across all four DENV. Primary infection induces cross-neutralizing antibodies to heterologous DENV serotypes which may be detected in the first few weeks following the acute phase of infection [7] however heterotyoic neutralizing antibody responses have been shown to decline over time [30,31]. Several months after repeat infection with heterologous DENV the breadth of cross-neutralizing responses has stabilized, and the profile of multi-serotype responses is likely maintained for life. The monotypic anti-DENV responses identified in the present study likely reflect a true single DENV infection, whereas the multitypic responses suggest a history of infections with at least 2 DENV serotypes. Our results suggest transmission of at least three DENV serotypes along the northern coast of New Guinea in the years prior to 1962.

The mountains of the New Guinea Highlands, which reach heights of almost 5000 meters, bisect New Guinea island and separate the northern and southern lowlands and coastal areas. The rugged terrain of the island has contributed to the development of geographically isolated population groups who speak more than 800 distinct languages. Interaction between groups up until the second half of the 20^th^ century was generally limited [32,33], and this may have restricted the introduction and transmission of acute viral diseases which require relatively close human contact. In addition, continuous contact with colonial authorities and other foreign forces did not occur until around the middle of the 20^th^ century for many groups; the first permanent Dutch government post and trade operations were established among the Asmat peoples, sampled in the present study, on the Casuarine Coast in 1953. At this time inter-group warfare had not ended and the fierce reputation of the Asmat people [34] coupled with the difficult coastal marshland terrain contributed to isolation of the local populations. Adels and Gajdusek [33] assessed measles seroprevalence in this population and identified a virgin soil epidemic in 1961, supporting our finding that the apparent lack, or rarity, of dengue transmission along the southern coast was likely a consequence of the isolation of the Asmat villages; the lack of sustained contact with the outside world limited introduction of DENV from external sources as it did for measles. There are no jungle-dwelling non-human primates in West Papua or Papua New Guinea to act as a dengue reservoir and to sustain a sylvatic cycle that might occasionally spill over into humans, as has been recorded in neighbouring Malaysia [35–37].

In studies conducted during a DHF epidemic in Central Java in the late 1970s [38] *Aedes aegypti* and *Aedes albopictus*, trapped in the rural district of Bantul, were both shown to be competent vectors for DENV-1-4 isolated from DHF patients in Java. There are no published descriptions of *Ae*. *aegypti* or *Ae*. *albopictus* along the Casuarine Coast during the time of the present study, in 1962; in the earliest reports from New Guinea island *Ae*. *albopictus* was first identified on the northern coast in Jayapura in 1962 and Alexishafen in Madang Province in 1972, and on the southern coast in the Western Province of PNG along the southern Fly River coastal fringe in 1992 [39]. However, serosurveys conducted in the 1970s identified evidence of previous Chikungunya virus transmission along the Casuarine Coast [40]. Chikungunya and dengue are both vectored by *Ae*. *albopictus* mosquitoes and these findings suggest that the potential for dengue transmission existed at the time of sampling for the present study but that transmission did not occur. *Ae*. *albopictus* is considered to be a relatively inefficient dengue epidemic vector and has a short flight range [41] and these factors may have constrained the introduction of DENV into the geographically isolated people of the Casuarine Coast prior to 1962.

This study has shown that multiple dengue virus serotypes circulated in the northern coastal areas of West Papua and Papua New Guinea prior to 1963. It is not possible to identify when the viruses were introduced into New Guinea or where they originated; however, a large number of dengue-like illnesses occurred in US soldiers deployed in this area during the Second World War [15]. There are no reports describing similar illness occurring among local populations in this period and we cannot determine whether DENV was imported into northern New Guinea with troop movements, or if multiple serotypes were already circulating at that time and spread rapidly within susceptible troops. Villagers on the northern PNG coast may have interacted with peoples of neighbouring countries or with visitors from other regions more often compared with inhabitants of the Asmat and Casuarine coasts in the south, who were uniformly seronegative in the present study.

Despite the large number of dengue cases among US soldiers deployed along the northern PNG coast in the 1940s there are no reports of severe dengue among the soldiers. The lack of dengue surveillance in PNG in the decades since then has meant that incidence and prevalence data are lacking. Awareness of the disease may not be high throughout PNG but it is nevertheless surprising that severe dengue has not yet been identified in the more than six decades since DENV was first isolated from New Guinea, especially since our present findings indicate that residents of the northern New Guinea coast had experienced multiple DENV infections in the years prior to 1963.

Antibody preservation in serum samples stored for many decades is a major limitation of this study. Serum specimens analyzed in the present study were collected in New Guinea between 1959–1963. The samples were kept cool until they were transferred for storage at -15 deg C [42]; sera were eventually stored at -20 or -80 deg C in the Serum Archive Facility at Binghamton University in New York [43]. Sample integrity was previously demonstrated in studies assessing chloroquine resistance in Plasmodium falciparum in archived sera collected from East Sepik in 1961–1962 [44]. Immunoglobulin deterioration over more than five decades of transport and storage might reduce the sensitivity of our assays, and thus our findings may represent an underestimation of the true prevalence of anti-DENV neutralizing antibodies in the study areas.

Although DHF has never been reported in Papua New Guinea it occurs in West Papua, where the first reported DHF epidemic occurred in Jayapura in 1993–1994 on the northern coast [45], attributed to DENV-1, DENV-2, and DENV-3. Emergence of severe dengue is consistent with the apparent long-term transmission of multiple serotypes in this area identified in the present study, a similar pattern to that seen in other endemic settings. In contrast the first known DHF epidemic on the southern coast of West Papua occurred in Merauke in 2001 [46], attributed to DENV-3. Merauke is a town with an estimated population of 78,000 located on the southern coast of West Papua bordering PNG to the east and what is now the Asmat Regency to the west. An historical review of hospital records from 1995 to 2000 revealed no evidence of previous epidemic or sporadic DHF and the authors suggested that the outbreak represented the first instance of epidemic dengue virus transmission. There are no data on DENV transmission in the southern coastal areas of PNG east of Merauke, and no reports of DF or severe dengue, although dengue, including severe dengue, has been reported in the neighbouring Torres Strait Islands [19,47] which stretch from the southern coast of PNG to the northern coast of Australia.

The reasons for the apparent lack of severe dengue in PNG are unclear and require further investigation. The pathogenesis of severe dengue disease is not well understood though DHF incidence is clearly associated with sequential infection with multiple DENV serotypes [9,10], and in-vitro studies in humans have identified immune enhancement as a mechanism for DHF immunopathogenesis [27,48,49]. In this model DENV genetic heterogeneity resulting in B- and T-cell-specific epitope antigen variation, the sequence of DENV infections, host HLA profiles, and host genetic variation are among the major virus and host factors which influence clinical outcome. A long history of transmission of multiple DENV serotypes as suggested in our present study would predict a higher incidence of severe dengue than has been observed to date. Similar findings have been reported for Haiti [50] where extensive DENV transmission has been recorded in the absence of detectable severe disease.

In summary we have identified serological evidence for transmission of dengue along the northern coastline of New Guinea island as measured by neutralizing antibody to multiple DENV serotypes in adults up to 72 years of age, sampled between 1959–1963. These findings confirm that multiple DENV serotypes circulated in this region at different times in the decades prior to the sampling period and support the notion that dengue has been a significant yet neglected tropical disease in PNG for more than 70 years. Dengue burden needs to be quantified, and molecular epidemiological studies need to be undertaken to assess the origins and distribution of DENV genotypes so as to develop an understanding of disease transmission and pathogenicity in PNG, particularly in the context of emerging dengue vaccines.

## Supporting information

S1 Table Sensitivity and specificity of anti-DENV microneutralization (MN) test. (PDF)
