# Supplementary material for: Serological evidence for transmission of multiple dengue virus serotypes in Papua New Guinea and West Papua prior to 1963
Source: PLoS Negl Trop Dis. 2017 Apr 24;11(4):e0005488. doi: 10.1371/journal.pntd.0005488 (PMC5426789; doi:10.1371/journal.pntd.0005488)
Supplement: S1 Table — (PDF) [file pntd.0005488.s001.pdf]

	Group	n	DENV-1	DENV-2	DENV-3	DENV-4
1	Anti-DENV seronegative*	20	<10	<10	<10	<10
2	Standard antisera^:					
	Anti-DENV-1	1	10	<10	<10	<10
	Anti-DENV-2	1	<10	20	<10	<10
	Anti-DENV-3	1	<10	<10	10	<10
	Anti-DENV-4	1	<10	<10	<10	10
3	Anti-JEV	2	<10	<10	<10	<10
4	Anti-MVEV	2	<10	<10	<10	<10
5	Anti-KUNV	1	<10	<10	<10	<10
6	Multitypic infection#:					
	DENV-2; DENV-1	1	10	80	10	<10
	DENV-1;DENV-3;DENV-2	1	160	80	80	20
	DENV-2;DENV-1;DENV-4	1	80	320	40	20

* Individuals confirmed seronegative for anti-DENV by microneutralization and hemagglutination inhibition assay, with negative NS1 ELISA test. ^Standard DENV-1-4 antisera NIBSC 05/248 (National Institute for Biological Standards and Control [NIBSC], Potter's Bar, Hertfordshire, United Kingdom).# Individuals with well-defined (IgM/PCR positive) sequential DENV infection
